# GP0.4 from bacteriophage T7: in silico characterisation of its structure and interaction with *E. coli* FtsZ

**DOI:** 10.1186/s13104-016-2149-5

**Published:** 2016-07-13

**Authors:** Adam J. Simpkin, Daniel J. Rigden

**Affiliations:** Institute of Integrative Biology, University of Liverpool, Liverpool, L69 7ZB UK

**Keywords:** ab initio modelling, Homology modelling, Docking, FtsZ, Drug design

## Abstract

**Background:**

Proteins produced by bacteriophages can have potent antimicrobial activity. The study of phage-host interactions can therefore inform small molecule drug discovery by revealing and characterising new drug targets. Here we characterise in silico the predicted interaction of gene protein 0.4 (GP0.4) from the *Escherichia coli* (*E. coli*) phage T7 with *E. coli* filamenting temperature-sensitive mutant Z division protein (FtsZ). FtsZ is a tubulin homolog which plays a key role in bacterial cell division and that has been proposed as a drug target.

**Results:**

Using ab initio, fragment assembly structure modelling, we predicted the structure of GP0.4 with two programs. A structure similarity-based network was used to identify a U-shaped helix-turn-helix candidate fold as being favoured. ClusPro was used to dock this structure prediction to a homology model of *E. coli* FtsZ resulting in a favourable predicted interaction mode. Alternative docking methods supported the proposed mode which offered an immediate explanation for the anti-filamenting activity of GP0.4. Importantly, further strong support derived from a previously characterised insertion mutation, known to abolish GP0.4 activity, that is positioned in close proximity to the proposed GP0.4/FtsZ interface.

**Conclusions:**

The mode of interaction predicted by bioinformatics techniques strongly suggests a mechanism through which GP0.4 inhibits FtsZ and further establishes the latter’s druggable intrafilament interface as a potential drug target.

**Electronic supplementary material:**

The online version of this article (doi:10.1186/s13104-016-2149-5) contains supplementary material, which is available to authorized users.

## Background

Resistance to antibiotics has become a source of great public concern recently, compounded by the slow emergence of new antibiotics [1]. One possible solution may be phage therapy. Bacteriophages (referred to hereafter as phages) are among the simplest and most abundant types of microorganisms [2], and have been used therapeutically for close to a century. With the advent of antibiotics, the use of phages therapeutically has decreased in the western world. However, as resistance to antibiotics becomes a pressing issue, phages are once again being seen as a way to combat disease [3, 4].

There are several advantages to the therapeutic use of phages; they affect highly specific targets that minimise collateral damage [5], they can target bacterial strains resistant to antibiotics, and they can be used to supplement the effects of antibiotics [6]. Phages are, however, not without problems. For example, the delivery of phages is a major challenge, with the immune system presenting a large hurdle [3] and the use of phages as a delivery method requires accurate diagnosis of the disease-causing bacteria and therefore slows down treatment.

Study of phage-host interaction can also inform small molecule drug discovery by revealing new drug targets and pinpointing their weaknesses. Proteins in phages have naturally evolved to find effective methods to disrupt bacteria. Furthermore, in multi-protein complexes involved in processes such as cell regulation, small numbers of amino acids form hotspots which contribute most of the free energy during interactions [7]. By studying how phage proteins disrupt the protein–protein interfaces, we can identify potential hotspots and target them when designing drugs. Such drugs would dispense with the specificity limitation of the original phage since they can be designed to target broadly conserved bacterial mechanisms potentially rendering unnecessary the diagnosis of the specific pathogenic bacterium [8, 9].

Upon infection of bacteria, phages take over the host resources through the actions of proteins expressed early in the infection. One such protein from the *Escherichia coli* phage T7 is gene protein (GP) 0.4. GP0.4 directly inhibits the filamenting temperature-sensitive mutant Z division protein (FtsZ) of *E. coli* by preventing its assembly into protofilaments both in vivo and in vitro [10].

FtsZ is a tubulin homologue that plays a key role in the division of bacteria cells [11–13]. Much like tubulin, purified FtsZ binds and hydrolyses GTP [13]. GTP binding induces the FtsZ to polymerise into one of two polar protofilament conformations; straight or gently curved [11–15]. Between FtsZ monomers, an active site is formed which hydrolyses the GTP, and remains accessible to the GTP-rich cytoplasm [13, 15]. Therefore GTP binding can be rapidly restored allowing protofilaments to consist of mostly FtsZ-GTP subunits resistant to depolymerisation [13]. The FtsZ protofilaments associate laterally to form bundles or sheets [11, 13]. As the protofilaments bundle together they form the Z ring at the site of cytokinesis. Once assembled, the Z ring plays a crucial role in recruiting downstream proteins essential for cell division [12, 13]. Therefore the inhibition of FtsZ polymerisation prevents the division of the bacteria [10].

The exact mechanism through which GP0.4 interacts with FtsZ is unknown. However, a mutation in FtsZ that introduced a six nucleotide insertion (TCGGCG) overcame the toxicity of GP0.4 [10]. Here, using a suite of structural bioinformatics methods, we predict the structure of GP0.4 ab initio and determine a mode of interaction with FtsZ in accord with available data. The results suggest that the FtsZ protofilament interface is targeted in different ways by phage proteins for antimicrobial purposes or by the bacteria’s own proteins for regulatory purposes. These results add weight to the notion that this pocket is a druggable interface.

## Methods

### Sequence analysis

BLAST [16] was used with the GP0.4 and FtsZ sequences to identify homologous proteins in the UniProt database [17] and to search for any homologues already structurally characterised in the Protein Data Bank (PDB) [18]. The HHPred server was used to confirm that the structure of GP0.4 could not be inferred by distant homology to any known structure.

### GP0.4 ab initio modelling

To elucidate a structure for GP0.4, ROSETTA 3.5 AbinitioRelax modelling [19–24] was used. The AbinitioRelax application consists of two steps; the first step is a coarse-grained fragment-based search for conformation that uses a score function which favours protein-like features (Abinitio). The second step is an all atom refinement using the Rosetta full-atom force field (Relax). The Robetta server [22, 25] was used to generate the required fragments and the PSIPRED [26] secondary structure predictions used by ROSETTA.

Using ROSETTA, 10,000 ab initio models (or decoys) were produced for GP0.4 and clustered using default protocols on a Linux workstation. Representatives of the largest ten resulting clusters were considered as candidate fold predictions. This process was repeated for four homologues identified in the BLAST search. In addition to ROSETTA, GP0.4 and each homologous sequence was submitted to the QUARK ab initio server [27]. QUARK is an ab initio modelling method conceptually similar to ROSETTA. The server yielded a further ten models (representatives of the ten largest clusters) for each of the proteins.

### Validation and comparison of GP0.4 models

GROMACS 5.0.1 molecular dynamics [28–32] was used to test the stability of the models over a period of 5 ns using a cubic box filled with water as the solvent, chloride as the counterions, and the AMBER99SB-ILDN force field [33]. ProSA [34, 35] and QMEAN [36–39] were used to obtain protein structure quality measurements.

The top ten models produced for each protein by ROSETTA and QUARK were submitted to the ProCKSI comparative server [40] to assess any structural similarity between them by producing a matrix of template modelling (TM) scores [41]. This matrix was visualised using CLuster ANalysis of Sequences (CLANS) [42] to cluster the models with an attraction value of >0.6.

### FtsZ comparative modelling

ROSETTA comparative modelling (RosettaCM) [25] was used to make ten models of *E. coli* FtsZ based on ten homologues (2VAW, 2VXY, 4M8I, 1RQ2, 1OFU, 1W5F, 2VAP, 2R75, 3J4S and 4B45) obtained from HHPred [43–45]. RosettaCM optimises an all-atom energy function over the conformational space defined by homologue structures to produce models with more accurate side chain and backbone conformations than previously available. In order to select the best model produced, the inbuilt RosettaCM scoring system was used in combination with the model quality assessment program ProSA [34, 35].

### Docking

Four servers that performed well in the most recent CAPRI (Critical Assessment of PRediction of Interactions) competition [46] were used to predict how models of FtsZ and GP0.4 might interact—ClusPro [47–50], Swarmdock [51–53], Dock/PIERR [54] and GRAMM-X [55].

The final putative GP0.4 binding site was assessed for drugability with two complementary servers (DoGsiteScorer [56] and FTMap [57–59]) and its conservation between FtsZ sequences quantified with ConSurf [60–63].

## Results and discussion

ab initio modelling of GP0.4

**Fig. 1:**
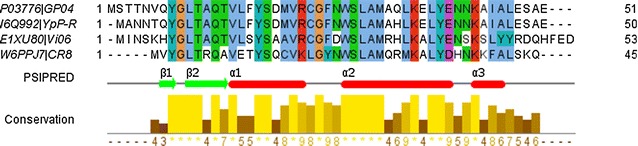
The alignment of GP0.4 (Uniprot accession: P03776), *Yersinia* phage YpsP-R (I6Q992), *Salmonella* phage Vi06 (E1XU80), and *Citrobacter* phage CR8 (W6PPJ7) using multiple sequence comparison by Log- Expectation (MUSCLE) [64, 65], and coloured using the Clustal colour scheme in Jalview [66]. The PSIPRED [67] secondary structure prediction for GP0.4 used in the ab initio modelling is shown below: *red bars* represent predicted α-helices and *green arrows* represent predicted β-strands

Since fragment-based ab initio modelling can benefit from consideration of homologous sequences, rather than focusing exclusively on the target, a BLAST [16] search with GP0.4 was done and identified four complete, non-redundant homologues in other phages (Fig. 1), none of which had been characterised.

**Fig. 2:**
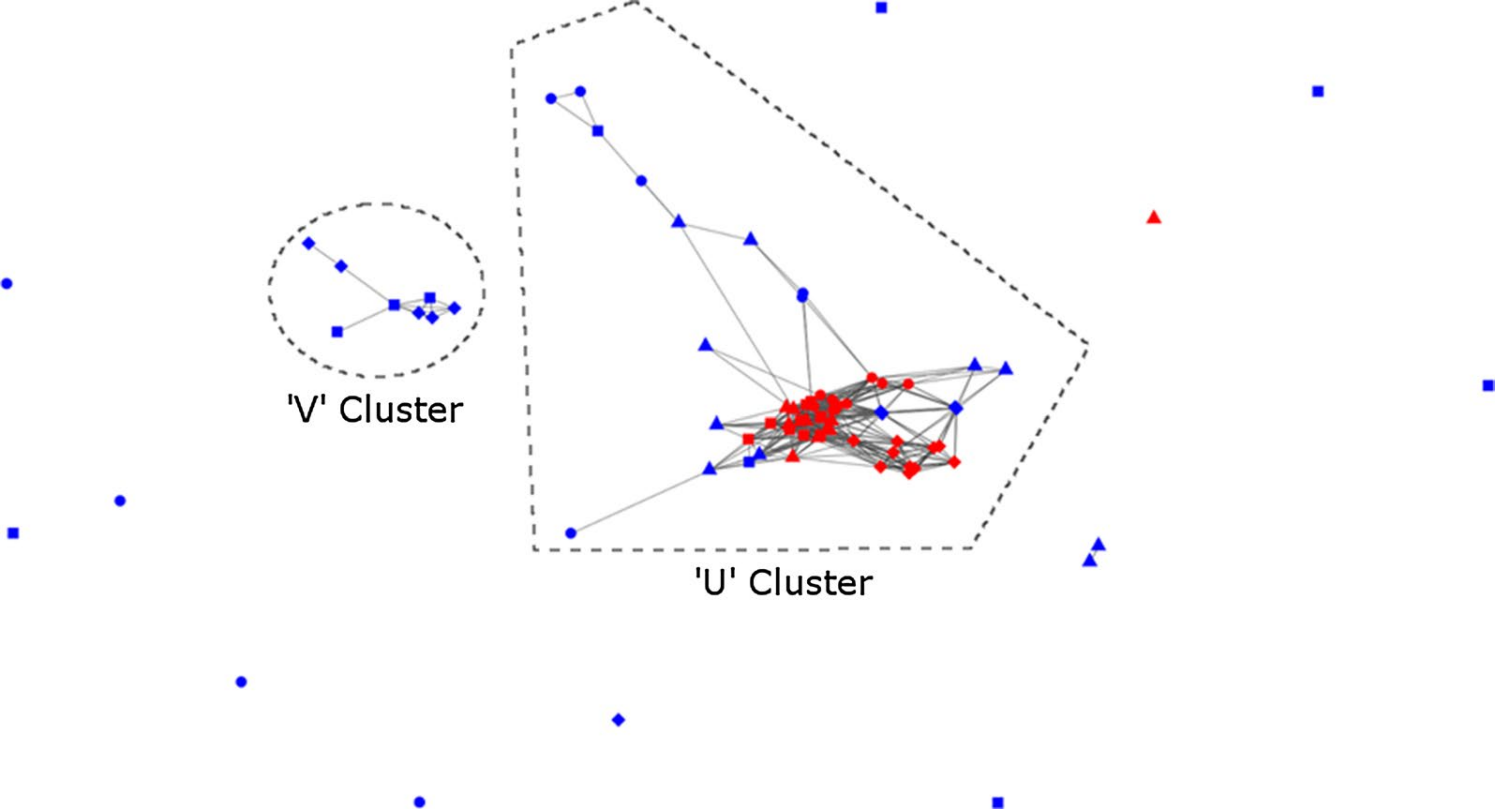
Clustering by pairwise TM scores of models of GP0.4 (UniProt accession number: P03776), *Yersinia* phage YpsP-R (I6Q992), *Salmonella* phage Vi06 (E1XU80), and *Citrobacter* phage CR8 (W6PPJ7). Each point represents a model, coloured by *origin blue* (Rosetta) or *red* (QUARK). *Shapes* indicate the sequence modelled: *square* GP0.4 (P03776), *triangle* YpsP-R (I6Q992), *circle* Vi06 (E1XU80) and *diamond* CR8 (W6PPJ7). *Lines* indicate TM scores of >0.6. The figure was made using CLANS [42]

Fragment-based ab initio modelling was done locally using ROSETTA [19–24] and remotely at the QUARK server [27] producing ten models (cluster centroids) each for GP0.4 itself (UniProt accession P03776), and homologues in *Yersinia* phage YpsP-R (I6Q992), *Salmonella* phage Vi06 (E1XU80), and *Citrobacter* phage CR8 (W6PPJ7). In order to look for consensus among the 100 model set, clustering was carried out based on pairwise TM scores obtained from the ProCKSI server [40]. TM scores are a well-established method to quantitatively measure the structural difference between two proteins [41] and range between 0 and 1: randomly chosen structures have an average score of 0.17 while a score of over 0.5 is considered as indicating that the two proteins have the same fold. Figure 2 shows GP0.4 and homologue models (nodes) linked by lines indicating a shared TM score of >0.6. This produced two clusters of note, one considerably larger than the other.

**Fig. 3:**
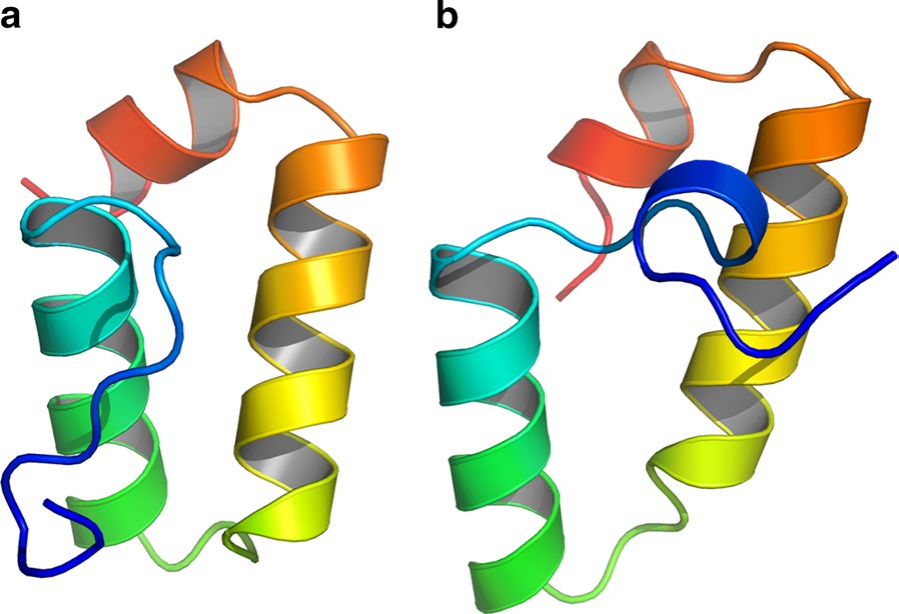
Comparison of GP0.4 models from the major (**a** ‘U’-shaped) and minor (**b** ‘V’-shaped) clusters in Fig. 2. Each is coloured from *N*-(*blue*) to *C*-terminus (*red*) as a spectrum. Visualised in PyMOL Molecular Graphics System, Version 1.7.4 Schrödinger, LLC. PyMOL was also used for Figs.  4, 5, 6 and 7

The larger cluster, containing ‘U’-shaped models was made up of QUARK predictions for GP0.4 and homologues from phages YpsP-R, Vi06 and CR8, along with Rosetta models for Vi06 and YpsP-R. The smaller and therefore less favoured cluster contained Rosetta models for GP0.4 and the CR8 homologue which were ‘V’-shaped. From each cluster, the GP0.4 model which made the most links to other structures was selected as a representative model for further analysis (Fig. 3). GROMACS [28–32] molecular dynamics was used to test the stability of these models and found both stabilize at low structural deviation (<1.75 Å rmsd on Cα atoms) from the starting structure within 5 ns. Similarly, model quality assessment with ProSA [34, 35] and QMEAN [36–39] did not favour one model or the other: within each cluster structures were found with ProSA scores as low as −4.36 and QMEAN scores as high as 0.734, within the range seen for experimental protein structures of a similar size. Furthermore, searches of the PDB using PDBeFOLD [68] and DALI [69] did not identify any significantly similar structures to either of the two GP0.4 models.

### FtsZ modelling

RosettaCM [25] was used to produce ten models of *E.coli* FtsZ using homologous structures from *P. aeruginosa* (1ofu, 2vaw), *M. tuberculosis* (1rq2)*, T. maritima* (1w5f)*, A. aeolicus* (2r75)*, M. jannaschii* (2vap)*, B.subtilis* (2vxy)*, B. thuringiensis* (3j4 s)*, H. volcanii* (4b45) and *S. epidermidis* (4m8i) as templates. FtsZ has a well conserved core domain followed by a variable *C*-terminal domain (Additional file 1: Figure S1). Indeed the ten models produced were very similar apart from that variable *C*-terminal domain which adopted a wide variety of poorly packed conformations. Previous studies of FtsZ–FtsZ interaction found that the *C*-terminal domain was not required for the assembly of protofilaments, but was essential for interaction with other membrane associated cell division proteins [13]. Since our focus here was on the inter-subunit interface of the protofilament targeted by GP0.4, the *C*-terminal region was eliminated and RosettaCM energy scores and ProSA [34, 35] scores were used to identify the most favoured model of the core domain.

### Docking

**Fig. 4:**
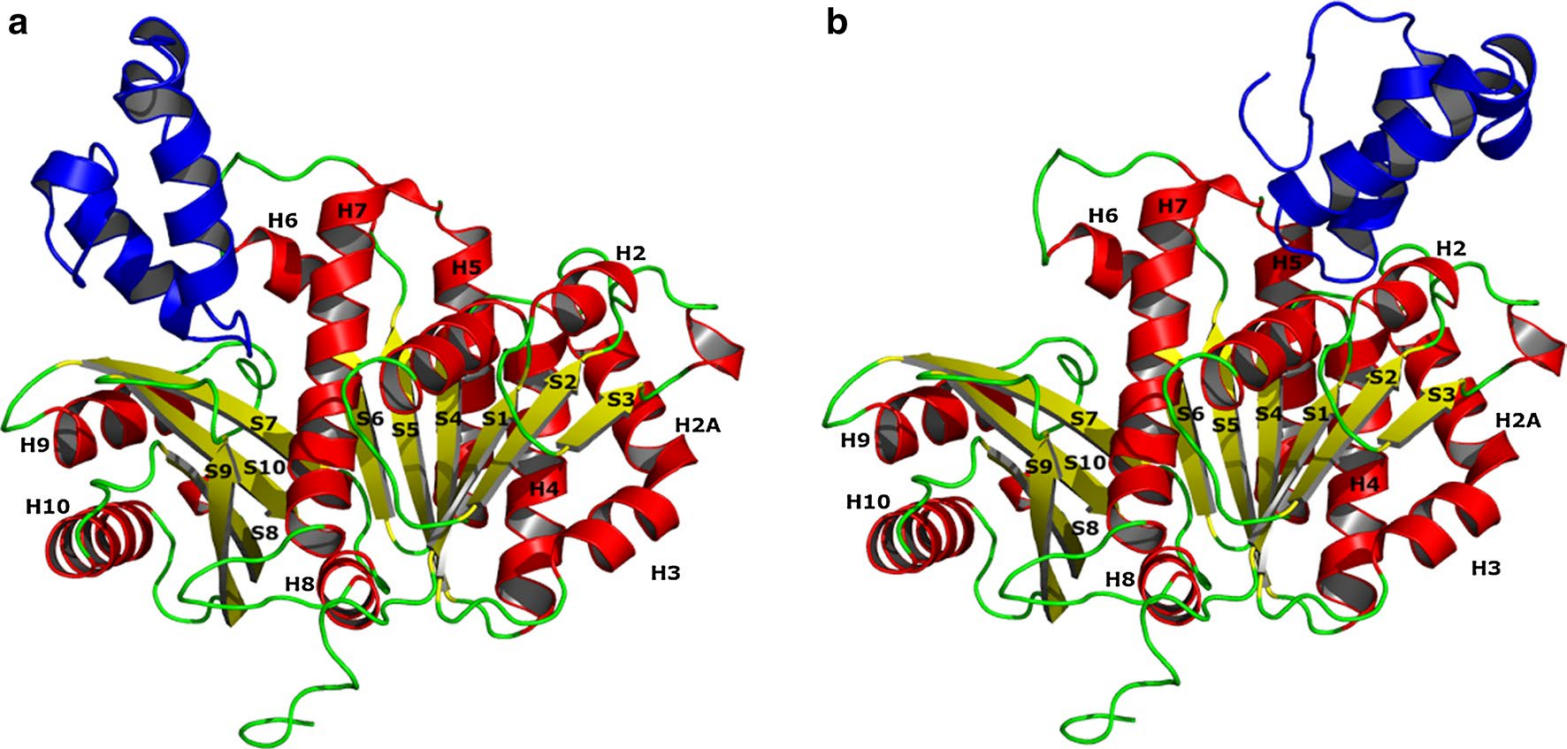
The top results when docking the ‘V’ form (**a**) and the ‘U’ form (**b**) of GP0.4 to the FtsZ model in ClusPro [47–50]. The GP0.4 model is coloured blue while the FtsZ structure is coloured by secondary structure: *red* for α-helices, *yellow* for β-strands, *green* for coil. Regular secondary structure elements in FtsZ are labelled H for α-helices or S for β-strands. The α-helices (H) and β-strands (S) shown in the cartoon representation of FtsZ are labeled according to the 3D structures of *Methanococcus jannaschi* FtsZ

Initial docking experiments between the GP0.4 ‘U’ and ‘V’ models and the FtsZ model were performed using the ClusPro server [47–50]. The ‘U’ and the ‘V’ model were predicted to bind to two different locations using default (‘balanced’) settings. The docking site for the ‘U’ model was obtained from a cluster of 280/1000 models, and the docking site for the ‘V’ model was obtained from a cluster of 382/1000 models. The ‘V’ model bound to a region flanked by H6, H7 and S7, whereas the more favoured ‘U’ model bound to a region flanked by H1, H5 and H7 (Fig. 4).

**Fig. 5:**
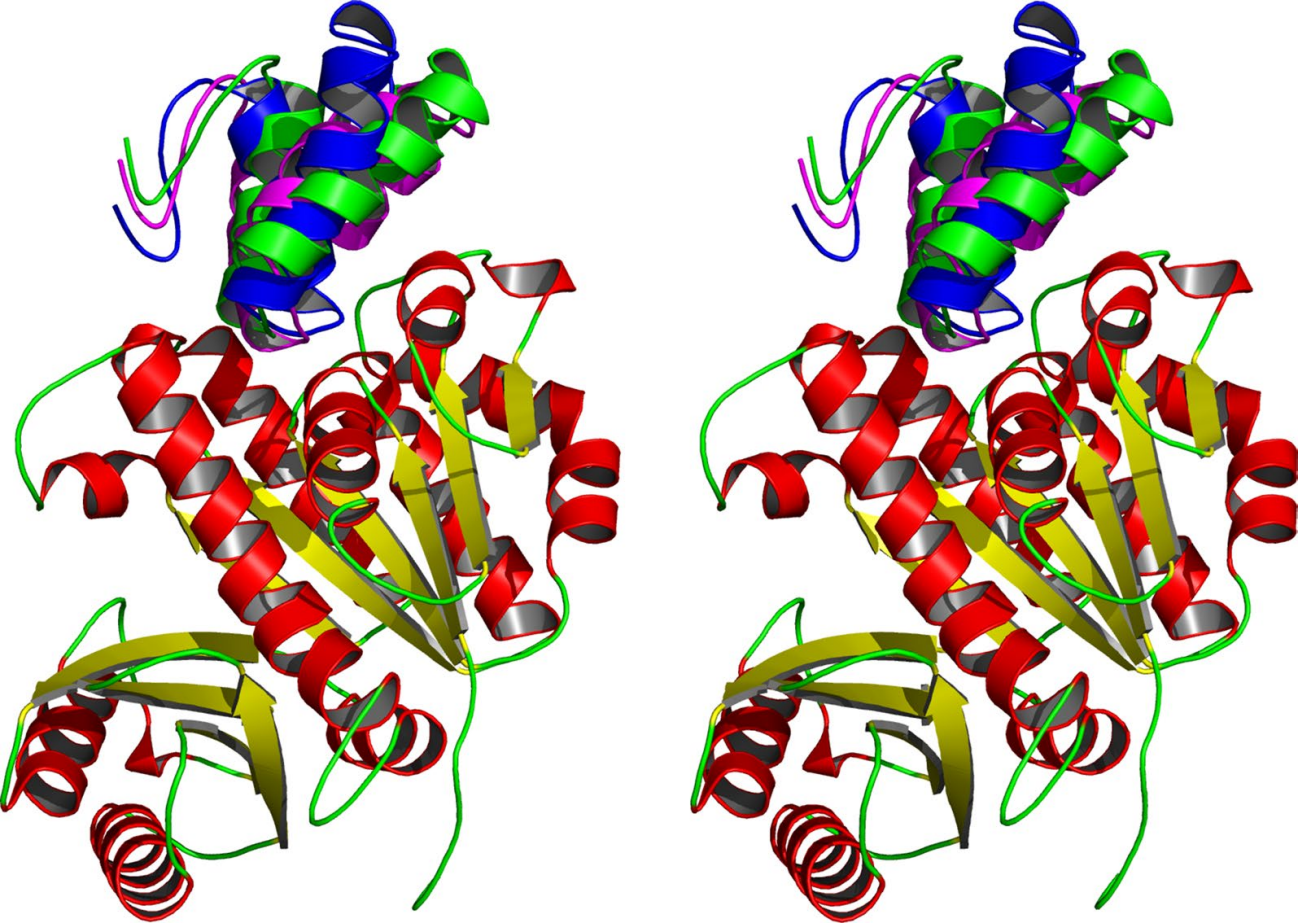
Cross-eyed stereo view of the consensus in docking of the ‘U’ form using ClusPro (*Blue*), Swarmdock (*Magenta*) and Dock/PIERR (*Green*). The FtsZ structure is coloured by secondary structure: *red* for alpha helix, *yellow* for beta sheet, *green* for coil

The results from ClusPro [47–50] were compared to those obtained using Swarmdock [51–53], Dock/PIERR [54] and GRAMM-X [55]. For the ‘U’ shaped model the top prediction from Dock/PIERR and the 6th highest prediction from Swarmdock were found to match the top prediction from ClusPro (Fig. 5). GRAMM-X also predicted that GP0.4 would bind to the same region of FtsZ but predicted a different docking pose (not shown).

**Fig. 6:**
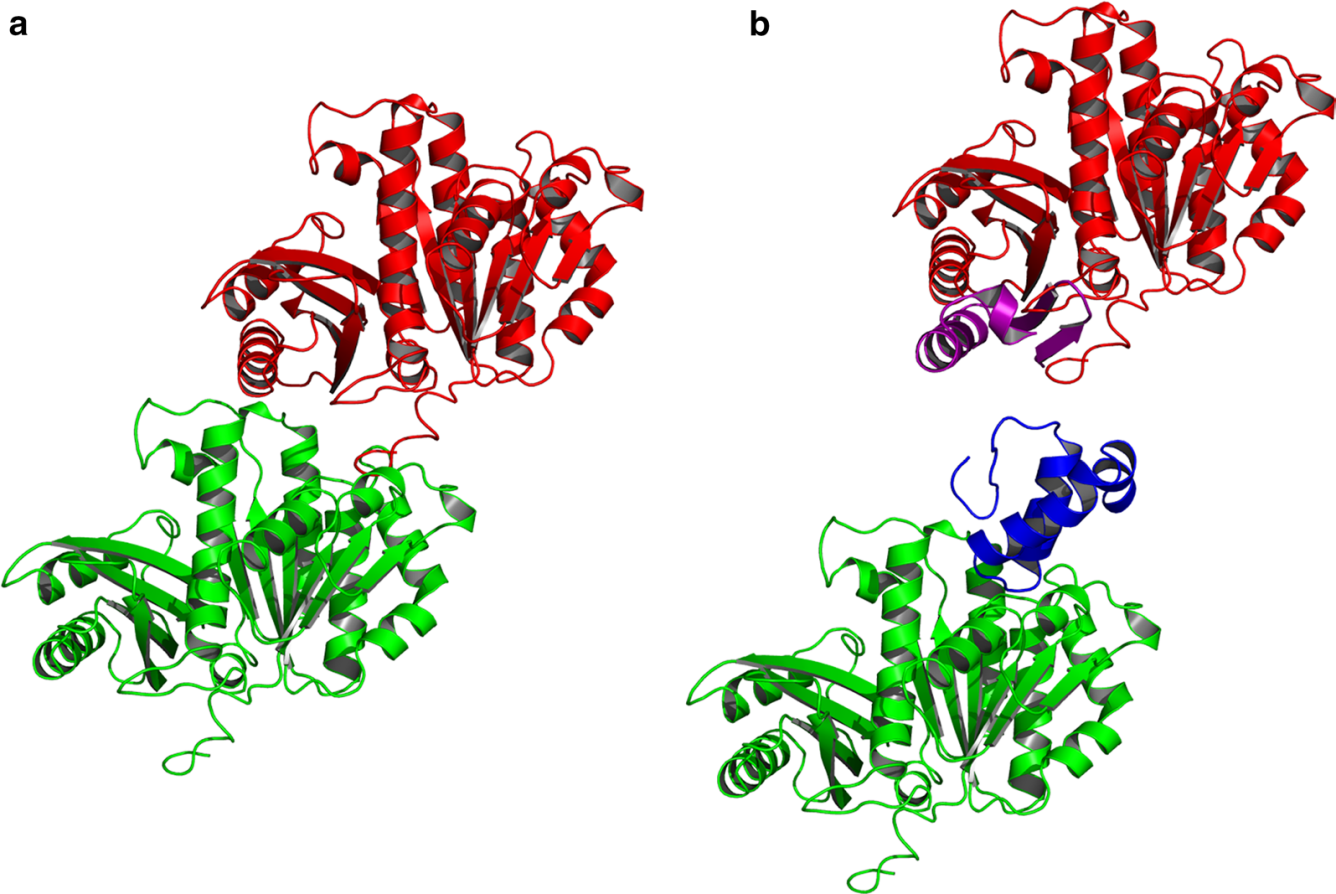
**a** The interaction between an upper FtsZ (*Red*) monomer and a lower FtsZ (*Green*) monomer in a protofilament. **b** the locations at which GP0.4 (*Blue*) binds to the lower FtsZ (*Green*) monomer and MciZ (*Purple*) binds to the upper FtsZ (*Red*) monomer. These can be seen to target the same region of the interface

In contrast, there was little agreement between the docking server results for the ‘V’ shaped model. For example, there was no coincidence between any of the top ten predictions from ClusPro, Dock/PIERR or Swarmdock. The lack of a consensus in the ‘V’ shaped model docking results is further indirect evidence supporting the ‘U’-shaped model since consistency between docking results would be expected of reliable results. Further support for the docking predictions for the ‘U’-shaped model is obtained by considering the FtsZ protofilament. The ‘U’-shaped model inserts its well-conserved central helix-turn-helix motif into a cleft between helices 1, 5 and 7 on FtsZ thereby preventing formation of a further intrafilament interface (Fig. 6). The conserved FtsZ residues G21, N24, G47 and G107 contribute to the binding by making hydrogen bond interactions with GP0.4. Given that the consensus ‘U’-shaped model offered such a plausible explanation for the anti-filamenting activity of GP0.4, attention was then focussed exclusively on it, excluding the ‘V’-shaped complex from further consideration.

### Mutant Ftsz modelling

**Fig. 7:**
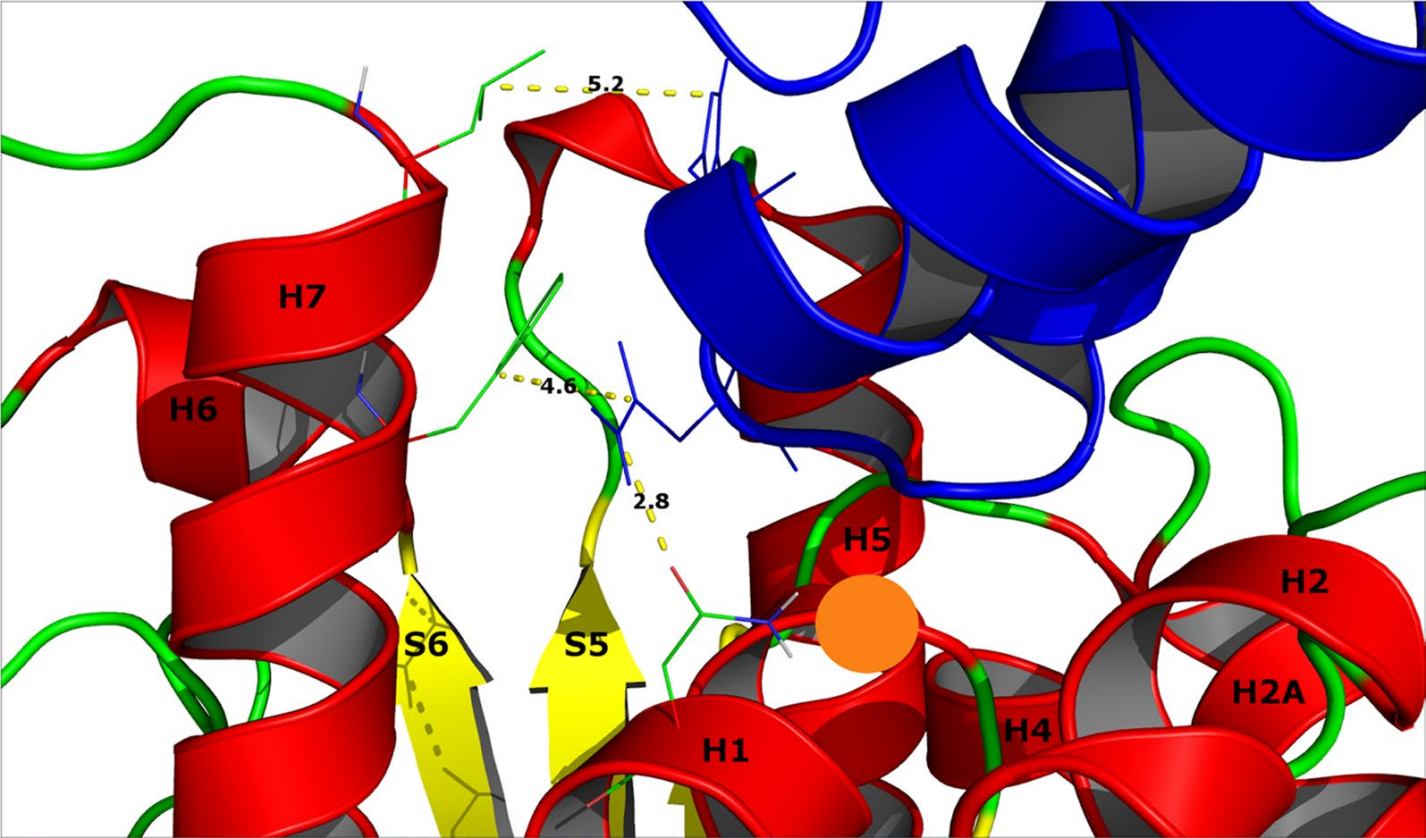
An enhanced view of the docked GP0.4. Interaction between GP0.4 (*blue*) and FtsZ residues N24 (2.8 Å), L178 (5.2 Å), and F182 (4.6 Å) are shown as *lines*. The *orange circle* indicates the location at where the mutation is inserted

Kiro et al. [10] identified a GP0.4 resistant variant of FtsZ, FtsZ9, where a 6 nucleotide insertion (TCGGCG) at position 18 resulted in a duplication of glycine–valine that allowed FtsZ9 to overcome the toxicity of GP0.4. It was reasoned that modelling this mutation might help identify how and where GP0.4 interacts with FtsZ. As shown in Fig. 7, the site of the mutation lies exactly at the base of the cleft to which GP0.4 is predicted to bind. This offers strong support to the binding model since the additional two residues encoded by FtsZ9 are thus perfectly placed to impeded GP0.4 binding and thereby confer resistance on the bacterium. The mutation also lies near the guanine nucleotide binding side of FtsZ, and the data of Kiro et al. [10] suggest that nucleotide binding is unaffected. However, we were unable to model a mutant FtsZ structure in which guanine nucleotides bound in the canonical fashion: conceivably, nucleotide binding is maintained in an altered fashion in FtsZ9.

Interestingly, MciZ, a developmental regulator of bacterial cell division was found to inhibit FtsZ polymerisation by targeting the same region of the intrafilament FtsZ interface as GP0.4 whilst binding to the opposite face of the FtsZ monomer [70], as shown in Fig. 6.

### Characteristics of the predicted GP0.4 binding site

**Fig. 8:**
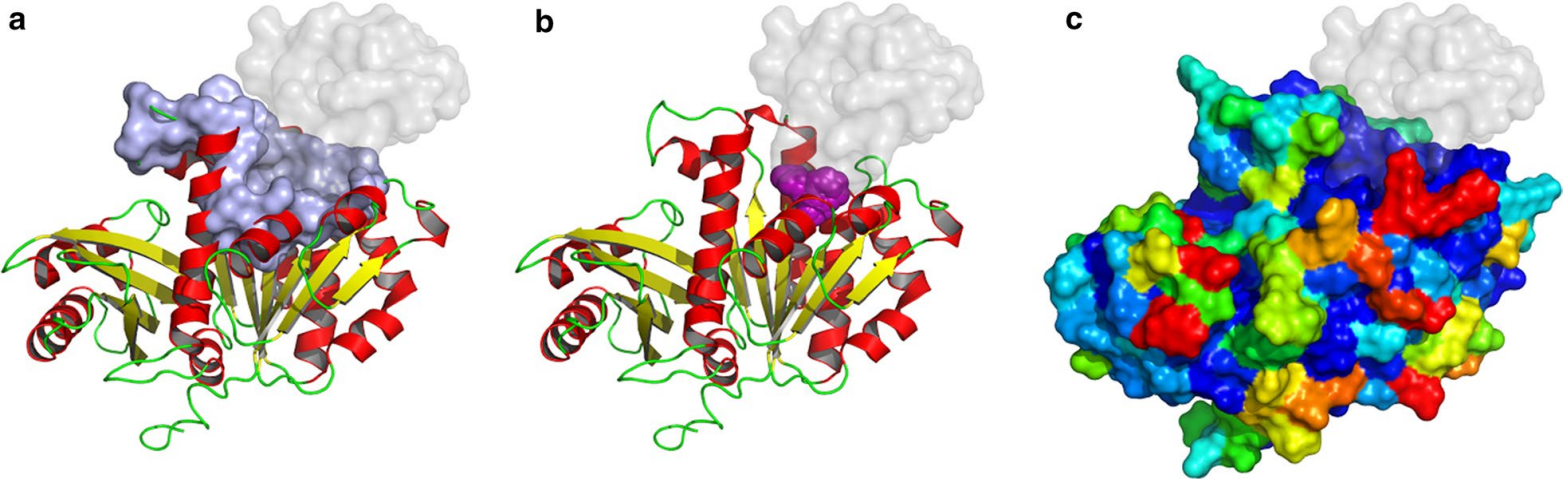
**a** The highest ranked drug pocket predicted by DoGsiteScorer (*mauve*) coincides with the putative GP0.4 binding site (GP0.4 surface shown as *transparent grey*). **b** The second highest ranked location of ligand binding predicted by FTMap (*magenta*) again coincides with the putative GP0.4 binding site (*grey*). In (**a**) and (**b**) FtsZ is coloured by secondary structure: *red* for alpha helix, *yellow* for beta sheet, *green* for coil. **c** ConSurf results indicating FtsZ amino acid conservation on a scale of *blue* to *red*, where *blue* is most conserved and *red* is least conserved

To help identify whether our putative GP0.4 binding pocket is a suitable candidate for small molecule drug design we used druggability servers; DoGsiteScorer [56] which looks for properties shared with known drug pockets and FTMap [57–59] which identifies areas bound by small organic probe molecules. The druggability servers both identified our pocket as a suitable biological target for drug binding (Fig. 8).

In order to assess whether the druggable predicted GP0.4 binding site is conserved among bacteria, we mapped sequence conservation of 150 FtsZ sequences onto the FtsZ structure using the ConSurf server. Figure 8 shows that the area the ‘U’ model bound to was well conserved. This provides evidence that a GP0.4 like protein or small molecule targeted to its binding site might be effective against FtsZ in other bacteria including pathogenic species: indeed, GP0.4 homologues were identified in phages for *Yersinia* and *Salmonella* supporting this idea.

The FtsZ filament interface targeted by GP0.4 has previously been highlighted as a possible target for small molecule targeting. However, there are concerns over targeting the GTP-binding site due to a risk of poisoning the eukaryotic homologue, tubulin [71]. Nevertheless, our results emphasise the druggable nature of a larger interface including the pocket targeted by GP0.4 They therefore encourage further efforts at exploiting the interface for small molecule drug design as well as offering possible routes forward for peptidomimetic inhibition.

## Conclusions

Characterising the targets and inhibitory mechanisms of phage proteins is a valuable route to the discovery and validation of new potential drug targets. Here we bring a range of structural bioinformatics methods to bear on the interaction between phage GP0.4 and its bacterial target FtsZ. We provide evidence that GP0.4 adopts a ‘U’ shaped conformation that inserts into a cleft between helices 1, 5 and 7 on FtsZ. The hypothesis is strongly supported by data obtained for a GP0.4-resistant FtsZ mutation [10]. The presence of GP0.4 bound to this region, as shown in Fig. 6, would physically interfere with assembly of the FtsZ filament. The importance of this FtsZ–FtsZ interface was further demonstrated by MciZ, a regulatory protein that binds to the opposite side of the interface to inhibit bacterial cell division. The druggable nature of the broad intrafilament FtsZ interface should encourage future drug design.

## Additional file

10.1186/s13104-016-2149-5 The alignment of FtsZ derived from *P. aeruginosa, B. subtilis, S. aureus, and M. tuberculosis* using MUltiple Sequence Comparison by Log-Expectation (muscle) [64, 65], and coloured using the clustal colour scheme. The alignment illustrates that *E.* *coli* FtsZ follows a known domain structure of FtsZ with a variable N-terminal segment (1–10), a highly conserved core region (10-316), a variable spacer (316–370) and finally a conserved C-terminal peptide (370–379) [13]. The figure was made with Jalview [66].
