# Supplementary material for: GP0.4 from bacteriophage T7: in silico characterisation of its structure and interaction with E. coli FtsZ
Source: BMC Res Notes. 2016 Jul 13;9:343. doi: 10.1186/s13104-016-2149-5 (PMC4944311; doi:10.1186/s13104-016-2149-5)
Supplement: Supplementary file 1 — 10.1186/s13104-016-2149-5 The alignment of FtsZ derived from P. aeruginosa, B. subtilis, S. aureus, and M. tuberculosis using MUltiple Sequence Comparison by Log-Expectation (muscle) [64, 65], and coloured using the clustal colour scheme. The alignment illustrates that E. coli FtsZ follows a known domain structure of FtsZ with a variable N-terminal segment (1–10), a highly conserved core region (10-316), a variable spacer (316–370) and finally a conserved C-terminal peptide (370–379) [13]. The figure was made with Jalview [66]. [file 13104_2016_2149_MOESM1_ESM.docx]

**Supplementary material**

**
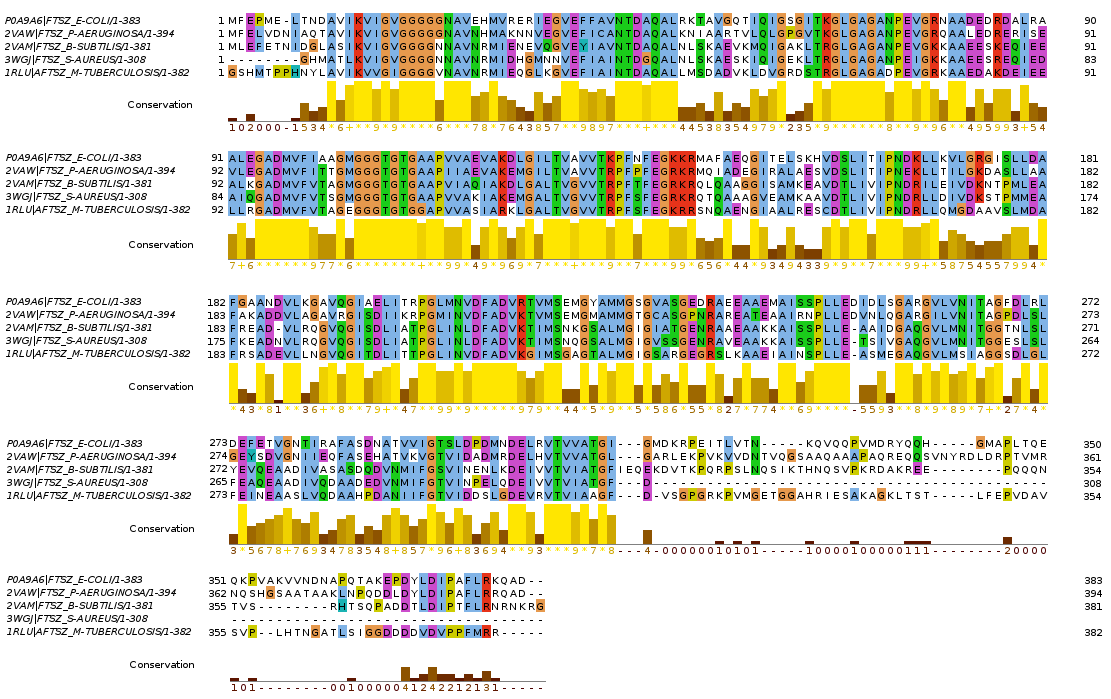
**

Supp. Fig. 1) The alignment of FtsZ derived from *P. aeruginosa, B. subtilis, S. aureus, and M. tuberculosis* using MUltiple Sequence Comparison by Log- Expectation (muscle) [1,2], and coloured using the clustal colour scheme. The alignment illustrates that *E.coli* FtsZ follows a known domain structure of FtsZ with a variable N-terminal segment (1-10), a highly conserved core region (10-316), a variable spacer (316-370) and finally a conserved C-terminal peptide (370-379) [3]. The figure was made with Jalview [4].

1. Edgar RC: MUSCLE: a multiple sequence alignment method with reduced time and space complexity. BMC Bioinformatics 2004, 5:113.

2. Edgar RC: MUSCLE: multiple sequence alignment with high accuracy and high throughput. Nucleic Acids Res 2004, 32:1792–7.

3. Margolin W: FtsZ and the division of prokaryotic cells and organelles. Nat Rev Mol Cell Biol 2005, 6:862–71.

4. Waterhouse AM, Procter JB, Martin DMA, Clamp M, Barton GJ: Jalview Version 2--a multiple sequence alignment editor and analysis workbench. Bioinformatics 2009, 25:1189–91.
